# Social perception in deaf individuals: A meta‐analysis of neuroimaging studies

**DOI:** 10.1002/hbm.26444

**Published:** 2023-08-23

**Authors:** Maria Arioli, Cecilia Segatta, Costanza Papagno, Marco Tettamanti, Zaira Cattaneo

**Affiliations:** ^1^ Department of Human and Social Sciences University of Bergamo Bergamo Italy; ^2^ Center for Mind/Brain Sciences (CIMeC) University of Trento Trento Italy; ^3^ Department of Psychology University of Milano‐Bicocca Milan Italy; ^4^ IRCCS Mondino Foundation Pavia Italy

**Keywords:** deaf, deafness, meta‐analysis, social cognition, social perception

## Abstract

Deaf individuals may report difficulties in social interactions. However, whether these difficulties depend on deafness affecting social brain circuits is controversial. Here, we report the first meta‐analysis comparing brain activations of hearing and (prelingually) deaf individuals during social perception. Our findings showed that deafness does not impact on the functional mechanisms supporting social perception. Indeed, both deaf and hearing control participants recruited regions of the action observation network during performance of different social tasks employing visual stimuli, and including biological motion perception, face identification, action observation, viewing, identification and memory for signs and lip reading. Moreover, we found increased recruitment of the superior‐middle temporal cortex in deaf individuals compared with hearing participants, suggesting a preserved and augmented function during social communication based on signs and lip movements. Overall, our meta‐analysis suggests that social difficulties experienced by deaf individuals are unlikely to be associated with brain alterations but may rather depend on non‐supportive environments.

## Graphical abstract

Deaf individuals may report difficulties in social interactions. However, whether these difficulties depend on deafness affecting social brain circuits is controversial. We found standard social brain activation in deaf individuals during communication based on signs and lip movements.

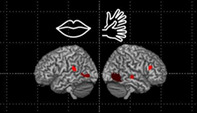

## 1 INTRODUCTION

Many are the positive effects of having successful social interactions (e.g., better quality of life, longevity, and health; Arioli et al., 2018; Kawamichi et al., 2016; Uchino, 2004). Natural spoken language is critical for social interactions, allowing to share thoughts, intentions, ideas, and emotions with other individuals (Hirsch et al., 2018; Tettamanti et al., 2017). Accordingly, regional neural activity and cross‐brain coherence in canonical language areas seem to be modulated by interpersonal interactions, as suggested by a study comparing brain activation during social interactions based on talking and listening (dual‐brain recording) compared to verbal noninteractive conditions (i.e., monologue) (Hirsch et al., 2018). Since auditory perception provides the basis for verbal communication (Kelsen et al., 2022), hearing impairments may raise a series of difficulties for social interactions mediated by speech (for a review, see Lemke & Scherpiet, 2015). Importantly, hearing loss is the most common sensory deficit worldwide, affecting more than half a billion individuals (Wilson et al., 2017). Deaf individuals typically rely on sign language, or hearing devices to interact with other people. Consistent evidence suggests that sign language supports the proper development of language brain circuits in deaf participants (for recent evidence, see Cheng et al., 2023; Wang et al., 2023) and sign language proficiency is associated with higher executive functions (e.g., performance in Go/no‐go task, Simon task; Kotowicz et al., 2023). Although it has been suggested that knowing sign language can help deaf individuals to feel comfortable and less stressed in social contexts (La Grutta et al., 2023), sign language can only be used with other signing individuals and hearing aids and cochlear implants (CIs) may not always work efficiently (e.g., Orji et al., 2020). This is especially in case of noisy environments, or when the other person is too distant or does not face the deaf person. These difficulties even increased during the COVID‐19 pandemic due to the detrimental effects on oral and signed communication of facial protection masks and preventive physical distancing (Al Majali & Alghazo, 2021; Almusawi et al., 2021; Giovanelli et al., 2023).

In light of the above, an interesting question is thus whether deafness impacts on the functional mechanisms and underlying brain circuits supporting social skills. Indeed, on the one hand, deaf individuals often report feelings of loneliness, social isolation (Bott & Saunders, 2021), and social exclusion (Alzuguren et al., 2019), especially in childhood and adolescence (Patel et al., 2021). On the other hand, available studies focusing on social abilities in deaf participants reported both compensatory (*enhancement hypothesis*, e.g., Bolognini et al., 2010; Ferrari et al., 2019) and impaired (*deficit hypothesis*, e.g., Sidera et al., 2017) mechanisms, suggesting the existence of a complex scenario that deserves further investigation (Cawthon et al., 2018). Some studies, indeed, showed that auditory deprivation is associated with difficulties in discriminating facial emotions (de Gracia et al., 2021), in representing others' beliefs and mental states (e.g., theory of mind; Figueroa et al., 2020), in empathy and prosocial motivation (Netten et al., 2015), possibly as a consequence of a lack of emotional disclosure, a reduced conversational activity, as well as a delay in language and narrative skills acquisition (Giustolisi et al., 2019). However, other studies suggest that deaf individuals, especially those that use sign language, may compensate for the lack of auditory input, performing as well as their hearing peers, or even better, in visual tasks tapping on social cognition, such as biological motion perception (Simon et al., 2020), face processing (Dobel et al., 2020), face emotion recognition (Ferrari et al., 2019; Krejtz et al., 2020), and face identity judgments (Letourneau & Mitchell, 2011).

Also at the neurophysiological level, mixed results are available regarding brain activation in deaf vs. hearing individuals during social cognition tasks. A neuroimaging study by Emmorey et al. (2010) reported diminished activation in the mirror neuron system in deaf signers, compared with hearing individuals, when they passively viewed some video clips of either signs or pantomime, compared to a fixation baseline. Accordingly, an earlier study reported that deaf signers presented less response compared with hearing individuals in the fronto‐parietal network associated with the mirror neuro system when passively viewing manual actions (Corina et al., 2007). Additional studies reported reduced responses in the temporo‐parietal junction in deaf participants, during mental state representation in a false belief task (Richardson et al., 2020). In turn, other studies observed stronger activation in the region encompassing the superior temporal sulcus and the superior temporal cortex (STC) in deaf versus hearing individuals in tasks requiring gesture recognition (Simon et al., 2020), facial expression discrimination (McCullough et al., 2005), action observation (Corina et al., 2007), and silent speech reading (Capek et al., 2008).

Hence, it seems that auditory deprivation may modulate social perception and social cognition tasks both at the functional and neural level. However, the available evidence is sparse and heterogeneous, and a systematic overview of the impact of deafness on social processing is not yet available. One reason is that deaf individuals are not a homogeneous group but may quite differ in terms of sign language use, hearing aids, CIs, onset of deafness and etiology (Fellinger et al., 2012; Pavani & Bottari, 2022). To shed light on the effects of deafness on social cognition and underlying brain circuits, we report here the first quantitative coordinate‐based meta‐analysis on published neuroimaging studies focusing on social perception in deaf versus hearing participants. Critically, this approach allows us to identify the brain regions consistently associated with social perception in condition of auditory deprivation, integrating the results of several experiments and overcoming the limitations inherent in single neuroimaging studies (e.g., sensitivity to experimental and analytic procedures, lack of replication studies, as well as small sample size; see Müller et al., 2018).

## 2 METHODS

### 2.1 Literature search and study selection

**FIGURE 1:**
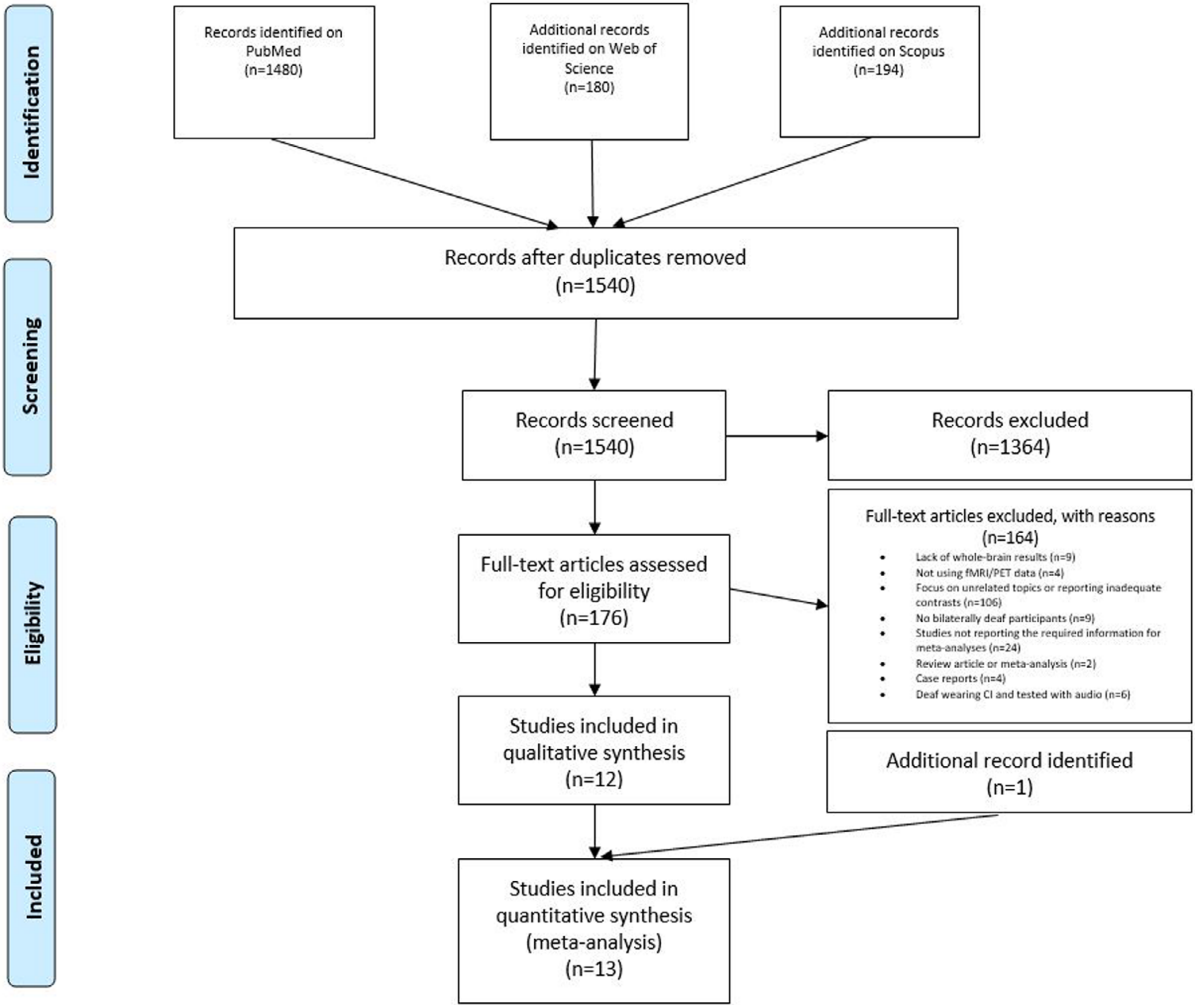
PRISMA flowchart (Moher et al., 2009) representing the study selection process.

The literature search and study selection procedures are described in Figure 1. Following current guidelines for meta‐analyses (Müller et al., 2018; Page et al., 2022), a literature search was performed on PubMed (https://pubmed.ncbi.nlm.nih.gov; research date: April 26, 2022, and updated in: January 19, 2023) using the following keywords: “deaf AND fMRI,” “deaf AND PET,” “deafness AND fMRI,” “deafness AND PET.” Filters were added, namely “Human” as the only species of interest, and “English” as the written language of target articles. Moreover, we performed a parallel search also on Web of Science (https://www.webofscience.com/wos/woscc/basic-search) and on Scopus (https://www.scopus.com/search/form.uri?display=basic#basic), using the same keywords and filtering for “articles” and “English.” After removing duplicates, we identified 1540 articles with this search.

We used the following inclusion criteria to select the articles of interest:

1. articles written in English;
2. use of functional magnetic resonance imaging (fMRI) or positron emission tomography (PET) neuroimaging technique to study brain activation;
3. whole‐brain analyses reported. Studies reporting region of interest and/or small volume correction analyses were in turn excluded;
4. brain activation data reported in Montreal Neurological Institute (MNI; Evans et al., 1993) or Talairach (Talairach & Tournoux, 1988) atlas coordinates;
5. results derived from univariate brain activation analysis and not from functional or effective connectivity analyses;
6. focus on social perception. Particularly, we aimed to highlight the neural bases associated with the representation of others, irrespective of stimulus types (e.g., videos, images, etc.) and tasks (e.g., attention, biological motion, memory, etc.). We selected studies reporting either: (a) comparisons between the representation of other individuals (i.e., social perception) and the representation of nonhuman entities (i.e., control condition without social perception); or (b) correlations between BOLD signal and performance in social perception tasks;
7. focus on bilaterally deaf participants;
8. results reporting within‐group simple effects. This allowed us to run within‐group (deaf individuals; hearing individuals) and between‐group (deaf individuals vs. hearing individuals) meta‐analyses;
9. focus on adult participants (i.e., age range between 18 and 65 years old);
10. studies with sufficient sample size (i.e., studies with less than five participants per group were excluded). Importantly, case report studies or studies with fewer participants were excluded because the results might be spurious and rarely replicated (for a similar approach, see Chen et al., 2018; Duda & Sweet, 2020);
11. studies combining fMRI or PET data acquisition with brain stimulation were excluded. Indeed, we were interested in considering neural activations only depending on specific tasks/conditions and not altered by either transcranial magnetic or electrical stimulation;
12. studies aimed to assess the efficacy of CIs in deaf individuals using a task requiring voice processing were excluded. Indeed, these studies were mostly carried out to assess sound perception restoration after the application of CIs in deaf subjects.

After title and abstract screening, 176 full‐text articles were further evaluated for eligibility. From this pool we excluded 158 articles for the following reasons: lack of whole‐brain results (*n* = 9); no fMRI or PET data (*n* = 4); focus on unrelated topics or report of inadequate contrasts (*n* = 106); no bilaterally deaf participants (*n* = 9); review article or meta‐analysis (*n* = 2); case reports (*n* = 4); studies not reporting the required information for meta‐analysis, such as brain activation coordinates (*n* = 22) and studies focused on deaf individuals wearing CIs and tested with auditory stimuli (*n* = 6). Thus, 12 eligible articles resulted from this selection procedure. We then expanded our search considering all studies quoting and quoted by each of these 12 articles. Moreover, to find other possible relevant articles, we evaluated all studies quoting and quoted by a review (Corina & Singleton, 2009) and by a meta‐analysis (Trettenbrein et al., 2021) on related topics. We also searched among the “similar articles” presented on PubMed for each of the articles identified. This second phase allowed us to find 1 more article, resulting overall in 13 articles fulfilling our criteria.

For the meta‐analysis on the deaf group, we included 14 experiments (described in 13 articles), with overall 464 activation foci and 167 deaf participants. In turn, in the meta‐analysis on the hearing group, we included 14 experiments (described in 13 articles), with overall 378 activation foci and 176 hearing participants. This numerosity is in line with Messina et al. (2021), van Veluw and Chance (2014), and Xiong et al.'s (2019) meta‐analyses on social processing in healthy individuals.

The study characteristics of the articles included in the two meta‐analyses are reported in Table 1. Table 2 presents relevant information pertaining to the deafness condition of the participants in each study. Importantly, all deaf participants were prelingually deaf. Indeed, in all participants deafness onset occurred before the age of 2 years (except for three participants that became deaf before the age of 4/5 years but were still classified as prelingually deaf by Trumpp & Kiefer, 2018, and by Benetti et al., 2017). All deaf participants were signers and most of them reported sign language as the primary language.

**TABLE 1:** Overview of the studies included in the two meta‐analyses.

*N*	First author(s) (et al.), year	Subjects	Deaf group: Mean age (years) and gender distribution	Matching variables	Imaging technique	Stimuli	Task	Contrast	Deaf group: Number of activation foci	Hearing group: Number of activation foci
1	Benetti et al. (2017)	Exp1: 15 deaf signers (LIS) and non‐signers; 16 hearing non‐signers	Mean age: 32.26; 7M and 8F	Age, gender, handedness and nonverbal IQ	fMRI	Images	One‐back identity task	Faces > houses	9	4
		Exp2: 15 hearing signers (LIS)	‐	Age, gender, handedness and nonverbal IQ	fMRI	Images	One‐back identity task	Faces > houses		9
2	Buchsbaum et al. (2005)	10 deaf signers (ASL)	Mean age: 38.3; 7M and 3F	‐	fMRI	Videos	View and rehearse signs	Signs > baseline	12	‐
3	Campbell et al. (2011)	12 deaf signers (BSL)	Mean age: 25.2; no gender information provided	‐	fMRI	Videos	Person and sign identification task	Social perception > baseline; correlation between BOLD and the performance on person identification task	17	‐
4	Capek et al. (2008)	13 deaf signers (BSL); 13 hearing non‐signers	Mean age: 27.4; 7M and 6F	Handedness, nonverbal IQ and gender	fMRI	Full‐color motion videos	Target‐detection task of silent words (speaker face visible)	Correlation between BOLD and TAS performance	10	5
5	Cardin et al. (2018)	Exp1: 12 deaf signers (BSL); 16 hearing signers (BSL)	Mean age: 25.7; 6M and 6F	Age and nonverbal intelligence with WASI	fMRI	Videos	Two‐back working memory task and control color task of point‐lights	Signs > objects	3	4
		Exp2: 16 hearing non‐signers	‐	Age and nonverbal intelligence with WASI	fMRI	Videos	Two‐back working memory task and control color task of point‐lights	Signs > objects	‐	2
6	Corina et al. (2007)	10 deaf signers (ASL); 10 hearing non‐signers	Age range: 20–29; 4M and 6F	Handedness	PET	Videos	Passive viewing of self‐oriented actions, transitive object‐oriented actions, and common ASL signs (visible performer)	Actions > baseline	54	59
7	Emmorey et al. (2010)	14 deaf signers (ASL); 14 hearing non‐signers	Mean age: 22.3; 7M and 7F	Education and handedness	fMRI	Videos	Attention task of ASL or pantomimes (visible performer)	Human perception > fixation	14	47
8	Okada et al. (2015)	13 deaf signers (JSL)	Mean age: 21.0; 7M and 6F	‐	fMRI	Visual stimuli	Memory task of Japanese kana letters, signs, and Arabic letters	Signs > texts	8	‐
9	Petitto et al. (2000)	Exp1: 5 deaf signers (ASL); 5 hearing signers (ASL)	Deaf group (*n* = 11; 5 deaf from Exp1 and 6 deaf from Exp2): mean age: 28; 6M and 5F	Handedness, proficiency of native language and education	PET	Visual stimuli	Linguistic task of meaningless non‐signs, meaningful signs	Signs > fixation	109	108
		Exp2: 6 deaf signers (LSQ); 6 hearing signers (LSQ)	Deaf (*n* = 11; 5 deaf from Exp1 and 6 deaf from Exp2): mean age: 28; 6M and 5F	‐	PET	Visual stimuli	Linguistic task of meaningless non‐signs, meaningful signs	Signs > fixation	153	105
10	Simon et al. (2020)	16 deaf signers and non‐signers; 16 hearing non‐signers	Mean age: 30.25; 5M and 11F	Age, gender and education	fMRI	Point‐light animated videos	Biological motion task of animated point‐lights	Biological motion > scrambled	6	6
11	Trumpp and Kiefer (2018)	18 deaf signers (GSL); 18 hearing non‐signers	Mean age: 43.7; 10M and 8F	Age, gender, handedness, educational background and nonverbal intelligence	fMRI	Videos	Action and sign language observation task of hand action videos	Human perception > baseline	49	8
12	Waters et al. (2007)	13 deaf signers (BSL); 13 hearing non‐signers	Mean age: 27.4; 7M and 6F	Handedness, gender and nonverbal IQ	fMRI	Visual inputs (videos, texts, and pictures)	Target‐detection task of fingerspelling, signed language, text, and picture (visible signer)	Human videos > baseline	14	7
13	Weisberg et al. (2012)	Exp1: 10 deaf signers (ASL); 10 hearing non‐signers	Mean age: 23; 4M and 6F	‐	fMRI	Black and white photos	Match‐to‐sample task of faces and houses	Faces > houses	6	10
		Exp2: 8 hearing signers (ASL)	‐	‐	fMRI	Black and white photos	Match‐to‐sample task of faces and houses	Faces > houses		4
		Total: 167 deaf; 176 hearing							Total: 464	Total: 378

*Note*: TAS (Mohammed et al., 2006) and WASI (Wechsler, 1999). We used the “‐” when the information was not provided in the original papers.

Abbreviations: ASL, American Sign Language; BLS, British Sign Language; BOLD, Blood‐oxygen‐level‐dependent; F, female; fMRI, functional magnetic resonance imaging; GLS, German Sign Language; IQ, intelligence quotient; JSL, Japanese Sign Language; LIS, Italian Sign Language; LSQ, Langue des Signes Quebecoise; M, male; PET, positron emission tomography; TAS, Test of Adult Speechreading; WASI, Wechsler Abbreviated Scale of Intelligence.

**TABLE 2:** Characteristics of the deaf participants in the studies included in the meta‐analysis. The table reports information on deafness onset and/or duration, hearing loss, the presence of hearing aids, cochlear implants, and the primary language of the participants.

*N*	First author(s) (et al.), year	Onset	Etiology	Hearing loss	Hearing aids/cochlear implants	Stimulus type	Primary language
1	Benetti et al. (2017)	Prelingual/birth (*n* = 14), between 0 and 4 (*n* = 1)	‐	Severe or profound	Hearing aids: No (*n* = 5); partial (*n* = 2), full (*n* = 8) Cochlear implants: ‐	Only visual stimuli, with no audio	LIS (*n* = 6), spoken (*n* = 3), spoken/LIS (*n* = 6)
2	Buchsbaum et al. (2005)	Prelingual/birth	‐	‐	Hearing aids: ‐ Cochlear implants: ‐	Only visual stimuli, with no audio	ASL
3	Campbell et al. (2011)	Prelingual/birth	Congenital deafness	Severe or profound	Hearing aids: Partial (*n* = 8), full (*n* = 4) Cochlear implants: ‐	Only visual stimuli, with no audio	BLS
4	Capek et al. (2008)	Prelingual/birth	Congenital deafness	Severe or profound	Hearing aids: ‐ Cochlear implants: ‐	Only visual stimuli, with no audio	BLS
5	Cardin et al. (2018)	Prelingual/birth	Congenital deafness	Severe or profound	Hearing aids: ‐ Cochlear implants: ‐	Only visual stimuli, with no audio	BLS
6	Corina et al. (2007)	Prelingual/birth	‐	Profound	Hearing aids: ‐ Cochlear implants: ‐	Only visual stimuli, with no audio	ASL
7	Emmorey et al. (2010)	Prelingual/birth	‐	Severe or profound	Hearing aids: ‐ Cochlear implants: ‐	Only visual stimuli, with no audio	ASL
8	Okada et al. (2015)	Prelingual/birth	Congenital deafness	Severe or profound	Hearing aids: ‐ Cochlear implants: ‐	Only visual stimuli, with no audio	JSL
9	Petitto et al. (2000) exp1	Prelingual/birth	Congenital deafness	Profound	Hearing aids: ‐ Cochlear implants: ‐	Only visual stimuli, with no audio	ASL
	Petitto et al. (2000) exp2	Prelingual/birth	Congenital deafness	Profound	Hearing aids: ‐ Cochlear implants: ‐	Only visual stimuli, with no audio	LSQ
10	Simon et al. (2020)	Prelingual/early	Unknown (*n* = 12), congenitally deafness (*n* = 4)	Severe or profound	Hearing aids: Yes (*n* = 8) No (*n* = 8) Cochlear implants: ‐	Only visual stimuli, with no audio	LSQ (*n* = 8), spoken (*n* = 8)
11	Trumpp and Kiefer (2018)	Prelingual (*n* = 16), before age of 5 (*n* = 2)	Meningitis, unknown factors, otitis media, oxygen deficiency, pertussis	‐	Hearing aids: ‐ Cochlear implants: No	Only visual stimuli, with no audio	GLS
12	Waters et al. (2007)	Prelingual/birth	Congenital deafness	Severe or profound	Hearing aids: ‐ Cochlear implants: ‐	Only visual stimuli, with no audio	BLS
13	Weisberg et al. (2012)	Prelingual/birth	Congenital deafness	Severe or profound	Hearing aids: ‐ Cochlear implants: ‐	Only visual stimuli, with no audio	ASL

*Note*: We used the “‐” when the information was not provided in the original papers.

Abbreviations: ASL, American Sign Language; BLS, British Sign Language; GLS, German Sign Language; JSL, Japanese Sign Language; LIS, Italian Sign Language; LSQ, Langue des Signes Quebecoise.

The study selection was conducted by M.A. and C.S. The two researchers worked independently with regular meetings to clarify doubts. Eventually, the database was also reviewed and approved by all other authors. The activation foci (i.e., *x y z* coordinates reported in the experiments included in the two meta‐analyses) were extracted by C.S. and then checked by M.A. When an article reported the coordinates of activation before and after a clinical intervention, we only retained the coordinates of the baseline experiment, as recently suggested by Tahmasian et al. (2019). Since the inclusion of different experiments involving the same participants can decrease the validity of meta‐analytic results, when this was the case, we pooled the coordinates from all relevant contrasts as if deriving from just one experiment (Turkeltaub et al., 2002). This procedure was performed for 6 among the total of 13 articles.

### 2.2 Data analysis

Following the guidelines reported in Eickhoff et al. (2012) and Müller et al. (2018) for coordinate‐based meta‐analyses, we performed two activation likelihood estimation (ALE) analyses, using the GingerALE 3.0.2 software (Eickhoff et al., 2009), to identify regions consistently associated with social perception in, respectively, deaf and hearing participants.

First, activation coordinates reported in the Talairach atlas were converted to MNI atlas coordinates using the transformation tool implemented in GingerALE (icbm2tal function; Lancaster et al., 2007). The more conservative (i.e., smaller) anatomical mask size was used for all analyses.

Initially, each coordinate was interpreted as the center of a three‐dimensional Gaussian probability distribution, to capture the spatial uncertainty of each coordinate. Then, the GingerALE software combined the three‐dimensional probabilities of all coordinates for each voxel, thus creating a modeled activation (MA) map. To evaluate the convergence of results for each brain voxel, ALE scores were generated by the union of the MA maps (Turkeltaub et al., 2002). These ALE scores were compared to a null distribution (Eickhoff et al., 2012). In line with current recommendations (Eickhoff et al., 2017; Müller et al., 2018; Tahmasian et al., 2019), we used a statistical threshold of a *p* < .05 for cluster‐level with family wise error (FWE) rate correction (1000 permutations), with a *p* < .001 cluster‐forming threshold.

We then explored the common and different brain activation between deaf and hearing participants during social perception, performing a comparison between the two meta‐analyses, again using the GingerALE software. For this comparison, a conjunction image (i.e., deaf and hearing individuals) as well as two contrast images (i.e., deaf individuals > hearing individuals; hearing individuals > deaf individuals) were created. As inputs, we entered the ALE images resulting from the separate meta‐analyses on deaf and hearing participants. GingerALE randomly divided the foci of the two original datasets in two new datasets, preserving their sizes. For each of the new dataset, an ALE image was created, which was then subtracted from the other one and compared with the original data. The significance level for the contrast analysis was defined at an uncorrected threshold *p* < .001, with 1000 permutations and a minimum cluster size of 20 mm^3^. Importantly, there is no established method for multiple comparison corrections in contrast analysis in GingerALE: indeed, the only correction available is false discovery rate, but this option is marked with a “not recommended” indication, and the FWE correction is not available. Thus, for the contrast analysis, we did not adopt any corrections, as conventionally done (Eickhoff et al., 2011; for recent meta‐analyses using uncorrected threshold, see, e.g., Isherwood et al., 2021; Sacheli et al., 2022).

Brain localization of the significant meta‐analytic clusters was automatically generated by GingerALE (Eickhoff et al., 2012). For manual verification of the localizations, we used the SPM Anatomy Toolbox (v.2.2c; Eickhoff et al., 2005) and the AAL template (as implemented in MRIcron; https://www.nitrc.org/projects/mricron).

## 3 RESULTS

### 3.1 Social perception in deaf participants

**FIGURE 2:**
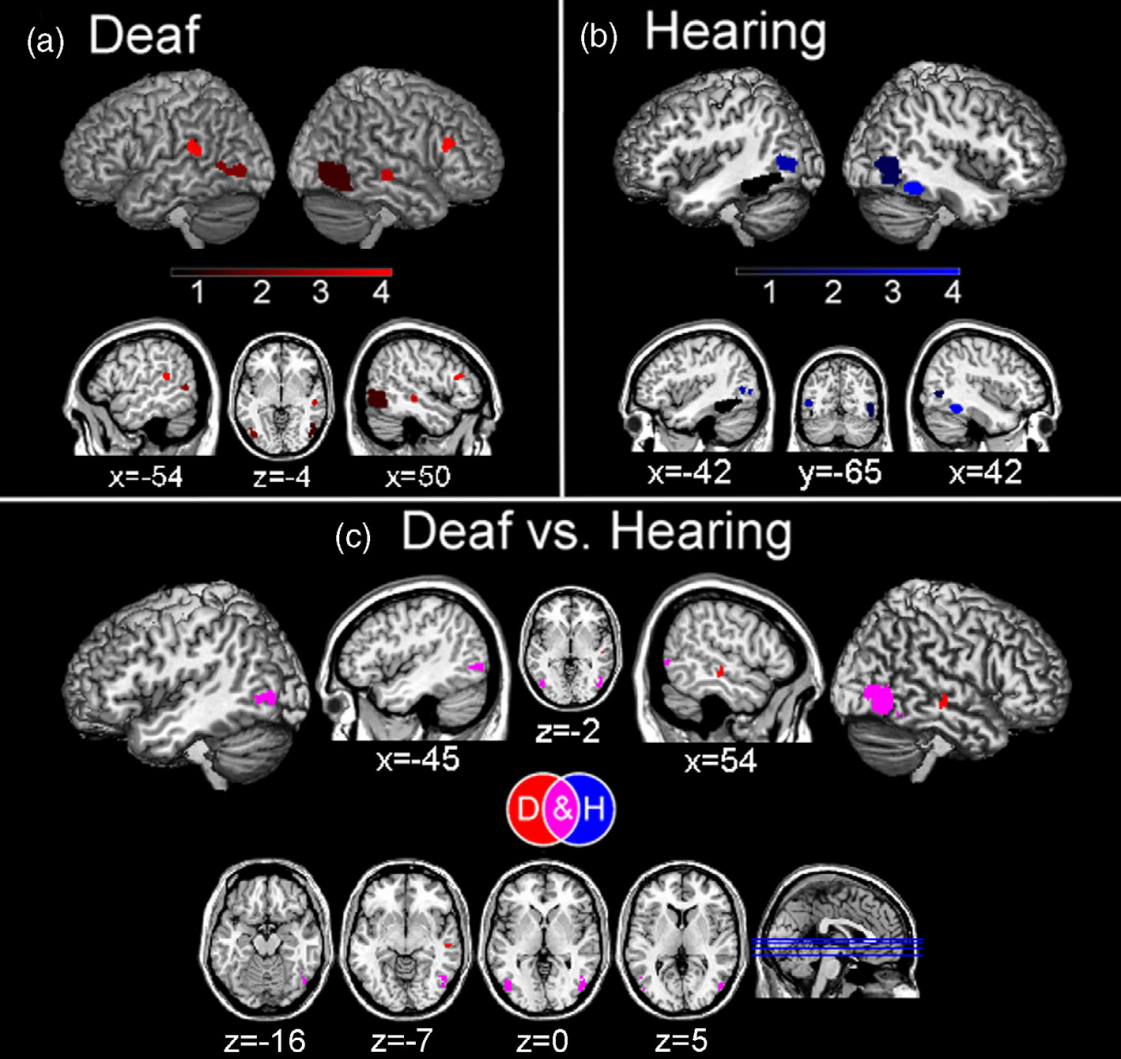
Brain areas consistently involved during social perception tasks in the deaf (a) and hearing (b) participants, and the specific and common activations between the two groups (c).

Overall, in deaf participants we found consistent meta‐activation during social perception tasks in the temporal cortex, specifically in the right inferior temporal cortex (ITC) as well as the middle temporal cortex (MTC) and STC bilaterally and the right fusiform gyrus (FG). Consistent activations were also found in the right inferior frontal cortex (IFC) and in the occipital cortex, specifically in the bilateral inferior occipital cortex and the right middle occipital cortex (Figure 2a and Table 3a).

**TABLE 3:** Brain areas consistently active in the deaf (a) and hearing (b) participants during social perception processes, as well as the overlapping (c) and different (d) activation between the two groups.

a. DEAF					
Cluster #	Volume (mm^3^)	*x*	*y*	*z*	Brain region
1	3952	50	−74	−2	Right inferior occipital gyrus/right middle occipital gyrus
“	“	48	−60	−10	Right inferior temporal gyrus/right fusiform gyrus
“	“	48	−60	−2	Right middle temporal gyrus
2	1488	−44	−76	−2	Left inferior occipital gyrus
“	“	−54	−62	2	Left middle temporal gyrus
3	880	54	−24	−6	Right middle temporal gyrus/right superior temporal gyrus
4	856	44	28	20	Right inferior frontal gyrus
5	832	−52	−42	14	Left middle temporal gyrus
“	“	−60	−36	20	Left superior temporal gyrus

b. HEARING					
Cluster #	Volume (mm^3^)	*x*	*y*	*z*	Brain region
1	3736	−40	−46	−18	Left fusiform gyrus
“	“	−42	−52	−16	Left fusiform gyrus
2	3184	48	−74	2	Right inferior occipital gyrus/right middle occipital gyrus
3	2144	−50	−68	4	Left middle temporal gyrus/left inferior temporal gyrus
4	1440	42	−48	−18	Right fusiform gyrus

c. DEAF and HEARING					
Cluster #	Volume (mm^3^)	*x*	*y*	*z*	Brain region
1		50	−74	0	Right inferior occipital cortex/right middle occipital gyrus
“	“	50	−64	−8	Right fusiform gyrus
2		−46	−72	0	Left inferior temporal gyrus
3		−52	−64	4	Left middle temporal gyrus/left inferior temporal gyrus
4		46	−56	−18	Right fusiform gyrus
5		48	−52	−18	Right fusiform gyrus

d. DEAF > HEARING					
Cluster #	Volume (mm^3^)	*x*	*y*	*z*	Brain region
1	368	54	−22	−8	Right superior temporal gyrus/right middle temporal gyrus

*Note*: We report for each cluster: number, volume size (in mm^3^), MNI coordinates (in mm), and anatomical labeling. Anatomical localization was derived from GingerALE (Eickhoff et al., 2012) and was checked using the SPM Anatomy Toolbox (v.2.2c; Eickhoff et al., 2005) and the AAL atlas (as implemented in MRIcron; https://www.nitrc.org/projects/mricron). For the two separate analyses, we adopted a cluster‐level *p* < .05 with FWE rate correction (1000 permutations), and a *p* < .001 cluster‐forming threshold. For the contrast analysis, we adopted an uncorrected *p* < .001 with 1000 permutations and a minimum cluster size of 20 mm^3^.

Abbreviations: FWE, family wise error; MNI, Montreal Neurological Institute.

### 3.2 Social perception in hearing participants

Hearing participants showed consistent activation in the bilateral FG, extending to MTC and to ITC in the left hemisphere. Moreover, we found consistent occipital cortex activation, in the right inferior and middle occipital cortex (Figure 2b and Table 3b).

### 3.3 Social perception in deaf versus hearing participants

We found common activation between the two groups in the right inferior occipital cortex and middle occipital cortex, as well as in the right FG and left ITC and MTC, suggesting that hearing is not necessary for the activation of these brain regions in social perception tasks (Figure 2c and Table 3c). While we did not find any stronger meta‐activation for hearing compared with deaf individuals in social perception tasks, we found that, compared to hearing, deaf participants had a stronger activation in the right MTC and right STC (Figure 2c and Table 3d).

## 4 DISCUSSION

To clarify the effect of auditory deprivation on social neurocognitive functions (e.g., Peterson, 2020), we conducted the first neuroimaging meta‐analysis on social perception in deaf versus hearing individuals. We found that during social perception tasks, deaf individuals recruited typical regions of the social brain.

Specifically, deaf individuals showed activation in an extensive occipito‐temporal network. This network encompasses the action observation network including the MTC (Hardwick et al., 2018) and the IFC (Urgen & Saygin, 2020), these regions supporting action observation and action recognition. Moreover, during social perception tasks deaf individuals recruited the STC, a region involved in mentalization processes and social information integration from both visual (Arioli et al., 2021b; Hirai & Senju, 2020) and auditory (Jiang et al., 2019; Yi et al., 2019) inputs. Finally, they showed a cluster activation extending through the inferior occipital cortex to the right FG, namely in regions that support face processing (Cohen et al., 2019; Kanwisher & Yovel, 2006; Palejwala et al., 2020; Volfart et al., 2022). These activations are consistent with what one may expect when considering the stimuli employed in the analyzed studies. In particular, several studies (a total of 10 studies, see Table 1) used visual stimuli representing bodies, gestures, and sign language, which likely led to the observed common activation in the MTC (de Gelder et al., 2015; Zhao et al., 2018). Activations in the FG possibly depended on several studies using stimuli depicting faces (3 studies used faces as stimuli, in other studies faces of the signing actors were visible; see Table 1), whereas activations within the action observation network were likely driven by stimuli representing, for example, actors performing signs and actions (used in 10 studies; see Table 1). Similar (but less extensive) activations in the occipito‐temporal network were also found in the meta‐analysis on social perception in hearing individuals. Hearing individuals, though, did not show specific activations of other brain areas known to be involved in social perception (e.g., superior temporal sulcus; Yang et al., 2015). This probably depended on the specific stimuli employed across the analyzed studies, such as sign language movements performed by visible signers (which were used in more than half of the included experiments; see Table 1), which may have been less socially salient for a hearing compared to a deaf person (e.g., Corina et al., 2007).

The conjunction meta‐analysis between deaf and hearing individuals showed a common activation pattern in the right inferior occipital lobe, particularly in the right FG. This common activation in the right FG is consistent with the fact that faces and bodies (typically recruiting the right FG; Harry et al., 2016) are socially salient stimuli, regardless hearing status. Critically, contrast analyses revealed that hearing individuals do not recruit any additional brain regions compared to deaf individuals. In turn, in deaf individuals, social perception tasks were more strongly associated with temporal lobe activation, particularly in the right STC. This region is involved in several key processes for social cognition, such as biological motion processing (e.g., Molenberghs et al., 2010), face perception (e.g., Schobert et al., 2018), audio‐visual speech processing (e.g., Venezia et al., 2017), and eye‐gaze processing (e.g., Hooker et al., 2003). The stronger activation of this region in deaf individuals, compared with hearing participants, might depend on the stimuli employed in the included studies, mainly assessing social perception through lip‐reading (e.g., Capek et al., 2008) and sign language (e.g., Okada et al., 2015; Trumpp & Kiefer, 2018). Such stimuli are a more salient source of social information for deaf than hearing individuals (Dole et al., 2017). Accordingly, a positive correlation between STC activity and lip‐reading ability in deaf individuals has been observed (Capek et al., 2008) and the superior‐MTC has been shown to be specifically involved in sign language processing (e.g., Campbell et al., 2011; for a recent meta‐analysis on sign language see Trettenbrein et al., 2021). Importantly, all deaf participants were signers and sign language represented their primary language (except for some participants in the study of Benetti et al., 2017 and of Simon et al., 2020; see Table 2).

Thus, in keeping with the *enhancement hypothesis*, our data seem to suggest a preserved and augmented function in the STC of deaf signers during social communication, based on the perception of signs and lip movements (Bottari et al., 2020). Importantly, successful social interactions in shared and reciprocal contexts have several implications in terms of quality of life and health (Kushalnagar et al., 2011) as well as for the typical development of cognitive function abilities (e.g., executive functions and language; Morgan et al., 2021) in deaf children.

## 5 LIMITATIONS

In considering our results, it is important to acknowledge that our meta‐analysis included a relatively small number of experiments, slightly below the number (i.e., 17) recommended by recent guidelines (Müller et al., 2018), but still in line with other recent meta‐analyses (Messina et al., 2021; Sacheli et al., 2022; Van Veluw & Chance, 2014; Xiong et al., 2019). However, we preferred to opt for a stringent selection criterion (including only prelingually deaf individuals, and excluding experiments performed with deaf individuals wearing CIs), and we are confident our results were not driven by a single or a few studies since we observed overall a strong convergence in the pattern of activations reported across the different experiments. Also, note that we employed an uncorrected threshold for comparison analysis (i.e., conjunction and contrasts): this is the standard accepted procedure when using GingerALE (see Eickhoff et al., 2011) that does not provide an established method for multiple comparison corrections in contrast analysis (for other meta‐analyses using this approach see Sacheli et al., 2022; Sokolowski et al., 2023). This is unlikely to have resulted into false positives in our study since our conjunction and contrast analyses only include clusters that have already passed the quite strict threshold of cluster‐level .05 and cluster‐forming threshold .001, used to create the single‐file maps (Sokolowski et al., 2023; for review see Eickhoff et al., 2012).

## 6 GENERAL CONCLUSION

Our results suggest that deafness does not prevent the development of the typical brain circuits underlying social perception, although social communication in deaf individuals needs to mostly rely on visual stimuli (e.g., signs and lip reading), without the support of auditory (i.e., voice) inputs. Critically, the same holds when considering blindness, that is a condition which also does not seem to interfere with development of critical circuits of the social brain (such as the mirror neuron system and the mentalizing system; Arioli et al., 2021a; Bedny et al., 2009; Ricciardi et al., 2009; Striem‐Amit et al., 2012). In addition, although evidence is rather scarce, it has been shown that tactile‐based communication in deafblind individuals is associated with an extensive brain circuit that involves typical nodes of the social brain, such as the STC (Obretenova et al., 2010). Taken together, these results suggest that the social brain is quite resistant to sensory deprivation, being in turn very adaptable and flexible (e.g., Bedny & Saxe, 2012; Setti et al., 2023; Voss et al., 2010), likely because it has been evolutionarily critical for survival (e.g., Dunbar, 2011; Insel & Fernald, 2004).

Our data suggest that in the presence of adequate social inputs, deaf signers activate the same brain circuits as hearing individuals, indicating a preserved social brain. Among these inputs, whether facial and bodily expressions, gestures, or postures are typically available for deaf individuals in social contexts, sign language is usually restricted to deaf communities. In light of this, promoting learning of sign language in hearing individuals, as well as providing salient visual cues in social situations, would facilitate social inclusion of deaf individuals (Jones et al., 2021). It is therefore particularly important to support the development of inclusive guidelines for everyday social contexts (e.g., Santos & Portes, 2019; for a practical guide in scholar settings, see Alasim, 2018; for work settings, see Foster & MacLeod, 2003).

## CONFLICT OF INTEREST

None of the authors have a conflict of interest to declare.

## Data Availability

All data can be found in the included articles.
